# Risk Factors Associated With de Quervain Tenosynovitis in Postpartum Women

**DOI:** 10.1177/15589447221150524

**Published:** 2023-01-24

**Authors:** Efrat Daglan, Samuel Morgan, Matan Yechezkel, Tal Frenkel Rutenberg, Shai Shemesh, Sorin D. Iordache, Assaf Kadar

**Affiliations:** 1 Tel Aviv University, Israel; 2 St. Joseph’s Health Care, London, ON, Canada; 3 Western University, London, ON, Canada

**Keywords:** de Quervain disease, postpartum women, tenosynovitis, hand surgery, risk factors

## Abstract

**Background:**

De Quervain (DQ) disease is caused by stenosis of the first dorsal compartment containing the abductor pollicis longus and extensor pollicis brevis. This condition affects women 6 times more than men and is also commonly reported in pregnant and lactating women. The natural course of the disease and associated risk factors are not well understood. In this study, we described the gestational risk factors associated with postpartum DQ.

**Methods:**

Sixty-three postpartum women with DQ were included in final study population. Medical records were reviewed for patient characteristics, including age, comorbidities, and body mass index (BMI), and gestational information, including length of pregnancy, gestation number, single or twin birth, and weight at birth. Odds ratio (OR) for developing DQ tenosynovitis were calculated with the control group of 630 postpartum women without DQ who gave birth between 2012 and 2020 in the same district.

**Results:**

Length of pregnancy (>40 weeks, OR = 5.81 [3.29-10.28]), first childbirth (OR = 2.23 [1.32-3.77]), and weight (BMI > 25, OR = 2.08 [1.14-3.81]) were all statistically significant risk factors associated with developing DQ. Number of fetuses > 1 (OR = 0.98 [0.29-3.33]) and birth weight more than 3.5 kg (OR = 0.60 [0.30-1.21]) were not associated with higher risk of DQ.

**Conclusions:**

Gestational risk factors associated with developing postpartum DQ include first pregnancy and long pregnancy of more than 40 weeks. Interestingly, child’s birthweight and number of fetuses, both factors that might increase load on the first dorsal compartment while holding the child, were not shown to increase the risk of postpartum DQ.

## Introduction

De Quervain (DQ) tenosynovitis is a disease involving the first extensor compartment of the hand, including tendons of the extensor pollicis brevis and abductor pollicis longus muscles. De Quervain is characterized by pain on the dorsal area of the hand and pain when performing ulnar deviation and flexion of the wrist.^1^ Initially, patients are managed nonoperatively through physical therapy, application of a brace, and steroid injections to the first compartment. When patients are unresponsive to these treatments or the disease recurs, surgical release of the first compartment is commonly performed.^2^

Over the years, many risk factors have been proposed for DQ tenosynovitis, including female sex, black race, and age greater than 40 years old.^3,4^ Pregnancy is well documented to have a significant association with a diagnosis of DQ.^5,6^ From a mechanical standpoint, in the postpartum period, the stress placed on surrounding tendons that occurs through flexion and ulnar deviation when caring for the baby results in inflammation to the tendons and tendon sheaths, which predisposes mothers to this pathology.^7-10^ The findings of several studies suggest that DQ is self-limited and resolves after cessation of lactation.^10^

Despite the established association between DQ and pregnancy, the natural course of the disease along with its epidemiology and associated gestational risk factors are poorly described in the current body of literature. Using a database from the largest health insurance organization in the country, we devised the following study to better understand, investigate, and describe gestational risk factors associated with a diagnosis of DQ in postpartum women.

## Methods

**Figure 1.**
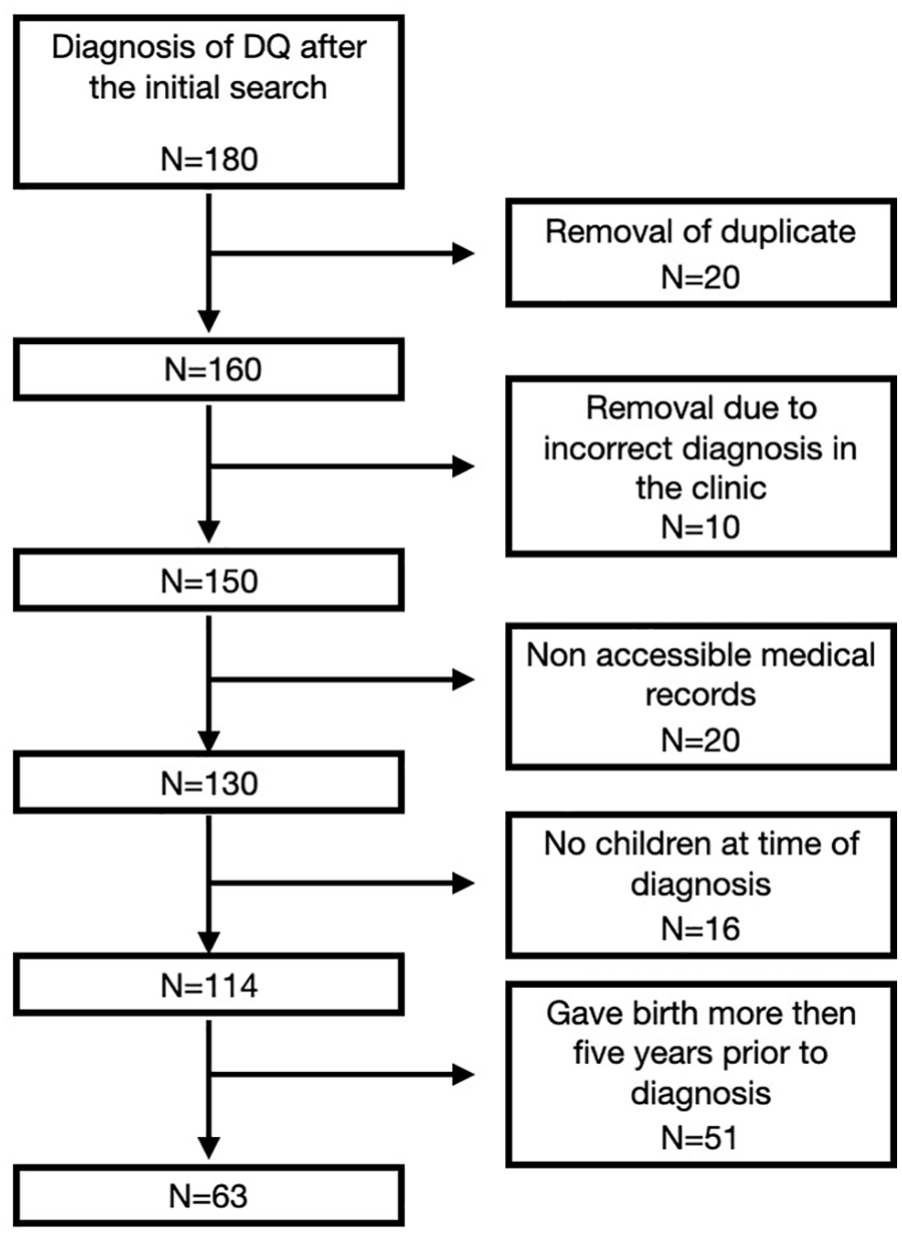
Cohort selection flowchart. *Note*. DQ = de Quervain.

A retrospective review was performed on postpartum patients with DQ treated by our service in a tertiary medical care center between 2012 and 2020. The study has been performed in accordance with the ethical standards in the 1964 Declaration of Helsinki and approved by the institution review board (RMC-20-0843). The study group was composed of women who were diagnosed with DQ for the first time and up to 1 year after giving birth. Excluded from our study were patients with other diagnoses, including carpel tunnel syndrome and carpometacarpal arthritis; patients without access to their medical records; and patients with a diagnosis of DQ who did not give birth in the year following their diagnosis (Figure 1). An age-matched control group of healthy women who gave birth during the same time period, in the same district, representing the same population in the area, was taken from the database of Clalit Health services, the largest public health insurance company in the country.

A diagnosis of DQ was made clinically, based on patient’s complaints, first compartment tenderness, and/or a positive Finkelstein test elicited on physical examination. When a differential diagnosis of thumb carpometacarpal arthritis was present, standard anteroposterior and lateral thumb radiographs were obtained (4 patients). An ultrasound was required in 13 patients to confirm the diagnosis of DQ.

Data extracted from the medical records of patients included characteristics such as age, comorbidities, body mass index (BMI), and smoking status at the diagnosis of DQ. In addition, information about the pregnancy and postpartum period was reviewed, which included length of pregnancy, gestation number, number of fetuses (single or twin gestation), and weight at birth.

### Statistical Analysis

For descriptive statistics, we presented mean with 95% confidence interval for continuous variables and presented categorical variables as numbers (percentages). Univariate analysis was performed with χ^2^ test for categorical data and Student *t* test for normally distributed continuous variables or Mann-Whitney for nonparametric data. A multivariate logistic regression was applied to identify the significant independent predictors for incurring DQ. We used the odds ratio (OR) to quantify the strength of association between exposure and disease. For each variable (exposure), we expressed the OR as the ratio of the number of cases to the number in controls in the exposed and nonexposed groups.

## Results

A total of 180 women with a diagnosis of DQ were identified from our search query. Following exclusions, the final case population for the analysis consisted of 63 patients (Figure 1). The control group consisted of 630 postpartum healthy women from the general population.

Of the 63 patients with a diagnosis of DQ, 3 (4.7%) were smokers, 4 (6.3%) were diabetic, 5 (7.9%) had a diagnosis of hypothyroidism, 3 (4.7%) had a diagnosis of asthma, 2 (3.1%) had a diagnosis of fibromyalgia, and 1 (1.5%) had a diagnosis of rheumatoid arthritis (Table 1). Fibromyalgia was the only comorbidity that was significantly higher in the patient’s population compared with the general population. Mean age of baby at onset of symptoms was 5.4 months. Mean duration of symptoms was 5.15 months.

**Table 1.** Patients’ Comorbidities.

Medical condition	Cohort (n = 63)	Control (n = 630)	*P* value
Smokers during pregnancy	3 (4.7%)	NA	NA
Diabetes mellitus	4 (6.3%)	15 (2.3%)	.096
Hypothyroidism	5 (7.9%)	26 (4.1%)	.20
Asthma	3 (4.7%)	11 (1.7%)	.13
Fibromyalgia	2 (3.1%)	0 (0%)	.009
Rheumatoid arthritis	1 (1.5%)	2 (0.3%)	.25
Inflammatory bowel disease	2 (3.1%)	4 (0.6%)	.10

*Note.* NA = Not applicable.

Key demographics found in our cohort included mean age of 33.7 years and BMI 25.3 (comparable with the control group). Total number of pregnancies was lower for women with DQ compared with the control group (DQ: 3, control: 3.61, *P* = .02). The average fetal weight of 3064.35 g was significantly lower than the weight of the control group (3222.58 g, *P* = .02). For patients with a diagnosis of DQ, the mean pregnancy week was 39.79 and was significantly higher than the control group (*P* < .001; Table 2).

**Table 2.** Demographic Characteristics of the Study and Control Group.

Characteristics	Sample size (cases)	Mean	95% CI	Sample size (control)	Mean	95% CI	*P* value
Age	63	33.70	32.24-35.16	630	33.83	33.40-34.26	.86
BMI	49	25.3	23.92-26.72	288	24.52	23.92-25.12	.11
Number of pregnancies	63	3	2.52-3.47	630	3.61	3.46-3.76	.02
Fetus weight, g	63	3064.35	2922.98-3205.72	630	3222.58	3182.66-3262.52	.02
Pregnancy week	63	39.79	38.91-40.68	630	38.58	38.43-38.73	<.001

*Note.* CI = confidence interval; BMI = body mass index.

Length of pregnancy (greater than 40 weeks) was the strongest risk factor for the development of DQ (OR = 5.81, 3.29-10.28). First child (OR = 2.23, 1.32-3.77) and BMI more than 25 (OR = 2.08, 1.14-3.81) were all factors that were found to have a statistically significant association with a diagnosis of DQ (Table 3). No significant association was noted between birth weight or twin birth and the development of DQ.

**Table 3.** Risk Factors for Postpartum de Quervain Disease.

Characteristics	Cohort (n)	Control (n)	Odds ratio (95% CI)	*P* value
First childbirth	34 (53.9%)	207 (32.8%)	2.23 (1.32-3.77)	.002
BMI > 25 (overweight)	24 (38%)	112 (17.7%)	2.08 (1.14-3.81)	.021
Number of fetuses > 1	3 (4.7%)	29 (4.6%)	0.98 (0.29-3.33)	.976
Pregnancy length > 40 weeks	25 (39.6%)	61 (9.6%)	5.81 (3.29-10.28)	<.0001
Fetus weight > 3200 g	25 (39.6%)	308 (48.8%)	0.61 (0.356-1.05)	.069
Fetus weight > 3503 g	10 (15.8%)	143 (22.6%)	0.60 (0.30-1.21)	.151

*Note.* CI = confidence interval; BMI = body mass index.

## Discussion

We devised the current study to determine gestational risk factors associated with a diagnosis of DQ in postpartum women. We found that first childbirth, length of pregnancy, and weight were associated with an increased risk of developing DQ. Surprisingly, twin birth and birth weight were not associated with increases incidence of DQ.

De Quervain disease is a common diagnosis in the postpartum period, resulting in pain, distress, and a worsened quality of life.^2^ The cause and pathophysiology of DQ is not well understood. Previous studies have proposed that repetitive motion of the first compartment of the hand^11^ and thickening of a tendon sheath through accumulation of mucopolysaccharide, an indicator of myxoid degeneration, contribute to the development of the pathology.^9,10^ Anatomical variation has additionally been proposed as a contributing factor in the pathophysiology of DQ. In a systematic review that evaluated anatomy within the first extensor compartment of patients with DQ and control cadavers, Lee et al^12^ noted significant anatomical variability in the first dorsal compartment, including the presence of accessory tendon slips and the existence of a septum in patients with a diagnosis of DQ.

**Figure 2.**
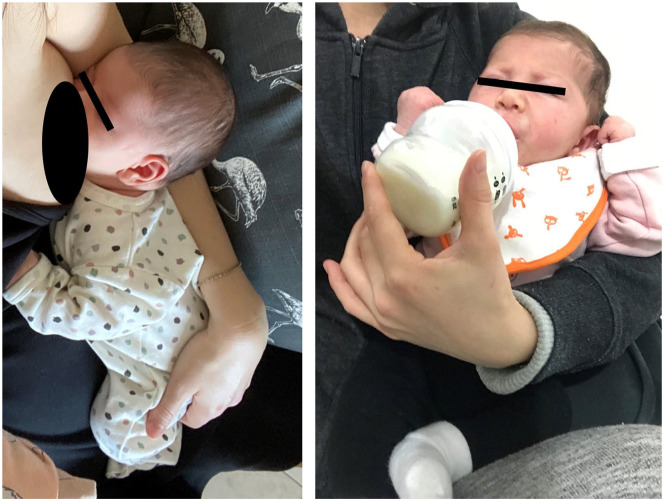
Flexed and ulnarly deviated wrist while breastfeeding or bottle feeding a newborn (informed consent obtained).

One of our study’s findings was that a first pregnancy was associated with a greater than 2-fold risk of developing DQ. We believe that such a finding may be attributed to several factors: (1) As this is a self-limiting condition, an experienced mother who already knows the course of the condition may not seek medical attention, understanding that her pain will resolve; (2) a mother who experienced DQ in the first pregnancy will likely have knowledge (ie, occupational therapy exercise) or equipment (ie, Brace), to help alleviate discomfort associated with this condition and thus, will not seek medical attention for her subsequent pregnancies; and (3) a less experienced mother will tend to place her wrist in the easiest position to support her child during lifting, breastfeeding, and bottle-feeding (ie, the wrist is in a position of maximal hyper flexion and slight ulnar deviation, supporting the buttocks of the baby, Figure 2). This position stretches the tendons in the first compartment which might result in DQ. The experienced mother, in her subsequent pregnancies, may choose to try different wrist positions to avoid this pain.

A long pregnancy (>40 weeks of gestation) had a 5.8 times higher risk of developing DQ. This association may be explained by edema of the first dorsal compartment.^13,14^ As the pregnancy progresses, the plasma volume increases, adding pressure to the first compartment. This may be further exacerbated by added fluid from breast feeding along with excess fluid from the third trimester that has not yet cleared. In addition, a high BMI of the mother increased the risk of incurring DQ in postpartum women. We believe that this finding may also be attributed to increased rates of edema in postpartum women that are overweight.^15^

This was the first study to investigate parameters associated with load to the wrist (birth weight and twin birth). We found that the newborn’s birth weight was not a contributing factor for the development of DQ, nor was twin birth. Intuitively, an increased load on the wrist is the main contributing factor for developing DQ. This finding however contradicts such a dogma. We hypothesize that the repetitive motion of the hand, such as in repeated lifting of the baby,^11^ in the hyperflex hyperulnar deviated position (similar to the Finkelstein position) has a more significant contribution to DQ than the force applied to the wrist.

This study is not without its limitations. First, this is a retrospective study, and results are limited by the quality and accuracy of data inputted. Some important variables such as baby’s weight at onset of symptoms were not reliably documented and therefore not included in the analysis. Second, our study was not powered to assess the effectiveness of the treatment for DQ. Third, lactation, which is a known risk factor for DQ, was not included in our analysis due to restrictions in the medical records that were charted. Despite these aforementioned limitations, we identified statistically significant risk factors associated with postpartum DQ that were not previously described in the literature.

## Conclusions

An overweight mother with a prolonged pregnancy and in her first pregnancy is at an increased risk of presenting with DQ disease. According to our findings, the weight applied to the carrying wrist (a heavy baby or twin babies) does not predispose mothers to this pathology. Physicians should readily consider these findings when educating and counseling patients with a diagnosis of DQ. Further well-designed studies are warranted to better understand factors that may predispose mothers to this pathology.
